# Optimization of Structure and Electrical Characteristics for Four-Layer Vertically-Stacked Horizontal Gate-All-Around Si Nanosheets Devices

**DOI:** 10.3390/nano11030646

**Published:** 2021-03-05

**Authors:** Qingzhu Zhang, Jie Gu, Renren Xu, Lei Cao, Junjie Li, Zhenhua Wu, Guilei Wang, Jiaxin Yao, Zhaohao Zhang, Jinjuan Xiang, Xiaobin He, Zhenzhen Kong, Hong Yang, Jiajia Tian, Gaobo Xu, Shujuan Mao, Henry H. Radamson, Huaxiang Yin, Jun Luo

**Affiliations:** 1 Advanced Integrated Circuits R&D Center, Institute of Microelectronic of the Chinese Academy of Sciences, Beijing 100029, China; zhangqingzhu@ime.ac.cn (Q.Z.); gujie@ime.ac.cn (J.G.); xurenren@ime.ac.cn (R.X.); caolei@ime.ac.cn (L.C.); lijunjie@ime.ac.cn (J.L.); wuzhenhua@ime.ac.cn (Z.W.); wangguilei@ime.ac.cn (G.W.); yaojiaxin@ime.ac.cn (J.Y.); xiangjinjuan@ime.ac.cn (J.X.); hexiaobin@ime.ac.cn (X.H.); kongzhenzhen@ime.ac.cn (Z.K.); yanghong@ime.ac.cn (H.Y.); tianjiajia@ime.ac.cn (J.T.); xugaobo@ime.ac.cn (G.X.); maoshujuan@ime.ac.cn (S.M.); rad@ime.ac.cn (H.H.R.); luojun@ime.ac.cn (J.L.); 2 Key Laboratory of Microelectronics Devices and Integrated Technology, Institute of Microelectronics, CAS, Beijing 100029, China; 3 School of Microelectronics, University of Chinese Academy of Sciences, Beijing 100049, China

**Keywords:** nanosheet (NS), gate-all-around (GAA), channel release, parasitic channel, suppression

## Abstract

In this paper, the optimizations of vertically-stacked horizontal gate-all-around (GAA) Si nanosheet (NS) transistors on bulk Si substrate are systemically investigated. The release process of NS channels was firstly optimized to achieve uniform device structures. An over 100:1 selective wet-etch ratio of GeSi to Si layer was achieved for GeSi/Si stacks samples with different GeSi thickness (5 nm, 10 nm, and 20 nm) or annealing temperatures (≤900 °C). Furthermore, the influence of ground-plane (GP) doping in Si sub-fin region to improve electrical characteristics of devices was carefully investigated by experiment and simulations. The subthreshold characteristics of *n*-type devices were greatly improved with the increase of GP doping doses. However, the *p*-type devices initially were improved and then deteriorated with the increase of GP doping doses, and they demonstrated the best electrical characteristics with the GP doping concentrations of about 1 × 10^18^ cm^−3^, which was also confirmed by technical computer aided design (TCAD) simulation results. Finally, 4 stacked GAA Si NS channels with 6 nm in thickness and 30 nm in width were firstly fabricated on bulk substrate, and the performance of the stacked GAA Si NS devices achieved a larger *I*_ON_/*I*_OFF_ ratio (3.15 × 10^5^) and smaller values of Subthreshold swings (*SS*s) (71.2 (N)/78.7 (P) mV/dec) and drain-induced barrier lowering (*DIBL*s) (9 (N)/22 (P) mV/V) by the optimization of suppression of parasitic channels and device’s structure.

## 1. Introduction

Three-dimensional (3D) fin field-effect transistors (FinFETs) have been widely used for the manufacture of high-volume integrated circuit (IC) products from 22 nm to 5 nm nodes because of their better channel electrostatic controllability and higher driving ability compared to those of conventional planar devices [1,2,3]. However, as the technology nodes scale down to 5 nm and beyond, many challenges, such as deteriorated electrostatic integrity, irresistible short-channel effects (SCEs), degraded device performance, and large process variability, appear for the FinFET structure [4,5]. Gate-all-around (GAA) Si nanowire/nanosheet (NW/NS) metal-oxide-semiconductor field effect transistors (MOSFETs) demonstrate a greater improvement in SCEs immunity than the conventional FinFETs due to the impact of much stronger control over the gate electrical field, and have been recognized as one of the most promising candidates beyond FinFFT technology [6,7,8,9,10]. In order to achieve a compatible fabrication approach with the mainstream FinFET process and improve the driving ability of the GAA NW/NS devices, stacked GAA Si NW/NS FETs have been proposed using conventional gate-last process, which provides a simple integration method by releasing NW channels from multilayer epitaxial GeSi/Si stacks in replacement high-k dielectric/metal gate (HK/MG) trenches [11]. However, compared with the traditional bulk FinFET architecture, the fabrication of stacked GAA Si NW/NS FETs suffers from a lot of challenges, such as NSs channel release, steep fin etch, inter-diffusion restriction of GeSi/Si stacks, inner spacers, and so on [12,13]. In addition, conventional techniques of parasitic sub-fin channel suppression, such as halo implantation for planar device and punchthrough stop (PTS) doping for bulk FinFET, are not suitable for GAA Si NW/NS devices, which need new approaches to reduce the leakage of parasitic sub-fin channel and improve device’s subthreshold characteristics [14].

In this paper, the optimization of NS release process of stacked GAA Si NS devices was carried out and optimal GAA Si NS channels and device structure was achieved. Furthermore, the influence of ground implantation (GP) doping on the electrical characteristics of devices was systematically investigated by experiment and technical computer aided design (TCAD) simulations. Due to the optimization of the fabrication process and device characteristics, good electrical properties were achieved.

## 2. Materials and Methods

**Figure 1:**
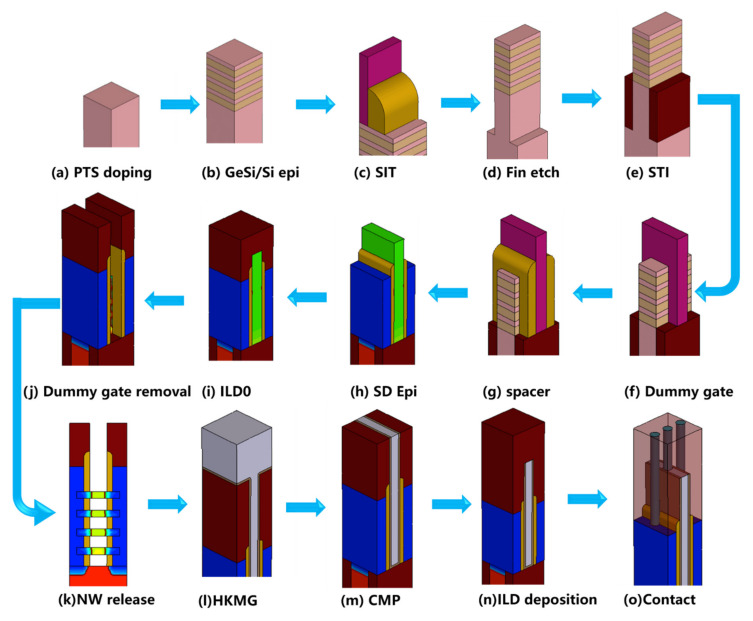
Fabrication flow of stacked gate-all-around Si nanosheet metal-oxide-semiconductor field effect transistors (GAA Si NS MOSFET): (**a**) 200 mm *p*-type (100) silicon wafers after ground-plane (GP) doping, (**b**) epitaxy of multi-layer GeSi/Si stacks, (**c**) the pattern of fin arrays formed by a spacer image transfer (SIT), (**d**) fin etch, (**e**) shallow trench isolation (STI) formation, (**f**) dummy gate formation, (**g**) Si_3_N_4_ spacers formation, (**h**) epitaxy of source drain (SD), (**i**) ILD0 deposition, (**j**) dummy gate removal, (**k**) Si NS channels release, (**l**) high-k dielectric/metal gate (HK/MG) formation, (**m**) chemical mechanical planarization (CMP), (**n**) ILD deposition, and (**o**) contact and metal.

The stacked GAA Si NSs MOSFETs were fabricated on 200 mm *p*-type bulk-Si (100) wafers with 8–12 Ω·cm resistivity. The integration process flow of the device is shown in Figure 1a, which is completely compatible with conventional bulk FinFETs with a relaxed pitch. In the first place, a GP doping process with 1 × 10^13^ cm^−2^, 1 × 10^14^ cm^−2^ and 5 × 10^14^ cm^−2^ doses (NMOS: B, 140 keV, 7°; PMOS: P, 140 keV, 7°) and no implantation (the reference devices) was carried out to investigate its influence on the device’s performance. In the following step, multi-layer GeSi/Si stacks with 12 nm Ge_0.3_Si_0.7_ and 10 nm Si were grown in a reduced pressure chemical vapor deposition (RPVVD) chamber (see Figure 1b). The patterns of fin arrays were formed by a spacer image transfer (SIT) technique [15,16] with a resolution over the advanced photolithography (see Figure 1c). The vertical fin with multilayer GeSi/Si stacks on the fin top were formed by advanced reactive ion etching (RIE) (see Figure 1d). In the following steps, a SiO_2_ with high aspect ratio process (HARP) was deposited, and a chemical mechanical planarization (CMP) was carried out to make the surface flat after a low temperature shallow trench isolation (STI) annealing. Then, a SiO_2_ etch back process was performed to reveal the fin by a diluted hydrofluoric (DHF) (see Figure 1e). A dummy gate stack with an ultra-thin oxide and thick amorphous Si (α-Si) was formed on the fins, which were patterned into nanoscale gate lines with an electron beam direct writing technique (see Figure 1f). The spacers were formed by deposition of thin SiN*_x_* and the RIE process (see Figure 1g). The Ge_0.3_Si_0.7_ epitaxy process with in-situ doping was carried out to reduce the parasitic resistance of SD and provide the strain to the channel (see Figure 1i). An optimized robust SiN*_x_* ILD0 material deposited by the low pressure chemical vapor deposition (LPCVD) approach was adopted to avoid micro-trench formation and gate spacer exfoliation. Then, a global planarization process was performed by chemical mechanical planarization (CMP), and the dummy α-Si gate lines buried in the ILD0 materials were exposed. The α-Si dummy gate was immediately removed by immersion in the tetramethylammonium hydroxide (TMAH) (see Figure 1j). The Si NS channels were formed by a selective wet-etch of GeSi (see Figure 1k). A DHF was applied for the removal of the native oxide before the formation of a high-quality interfacial (IL) by O_3_. The multilayer HK/MG film stacks were deposited by atom-layer-deposition (ALD) approach on whole NSs and surrounded the NS channels to form GAA structures (see Figure 1l). These complex process steps for the formation of a HK/MG by replacing the original dummy gate are together called the advanced replace metal gate (RMG) module. A global planarization process was performed by chemical mechanical planarization (CMP) to separate the gate. Finally, the gate and SD W-plug contact processes for the device were performed in the contact holes, and the whole device fabrication was accomplished by the formation of back-end metallization interconnection in the subsequent steps (see Figure 1o).

For the selective etch of GeSi in Ge_0.3_Si_0.7_/Si stack structures, 20 nm Si layers were sandwiched between 5 nm, 10 nm, and 20 nm GeSi layers, and Ge_0.3_Si_0.7_/Si stacks in equal thickness of 20 nm were epitaxially grown, annealed, and analyzed. The most uniform areas on the 200 mm epitaxial wafers were chosen by a thickness uniformity measurement. A conventional isotropic dry etch of GeSi/Si was carried out to form regular structures. The selected sample regions were then cut into 2 cm × 2 cm slices and annealed at different rapid thermal annealing (RTA) conditions between 600 °C to 1000 °C, respectively. GeSi/Si samples after different etching times in the mixed corrosion solution were laid up for 24 h (DHF (6%):H_2_O_2_ (30%):CH_3_COOH (99.8%) = 1:2:3).

The cross-sectional views and top views of the device’s structures were observed using S-5500 and S-4800 scanning electron microscopes (SEM, Hitachi, Tokyo, Japan), respectively. The cross-sectional profiles of the final device were performed using transmission electron microscopy (TEM, FEI Talos, Brno, Czech Republic) and energy-dispersive X-ray spectroscopy (EDX, FEI Talos, Brno, Czech Republic). The electrical characterization was performed using an Agilent 4156 (Agilent, Santa Clara, USA) semiconductor parameter analyzer. The 3D TCAD simulations were carried out by using Sentaurus TCAD tools. The device model was constructed by sprocess simulation and the schematic of baseline device structure was adopted according to the real fabricated devices. Fermi-Dirac statistics as well as quantum confinement effect, Hurkx Band-to-Band model low field ballistic mobility model, Shockley-Read-Hall, and Anger recombination models were implemented in the device simulation. 

## 3. Results and Discussion

### 3.1. Optimization of NS Release Processes of Stacked GAA Si NS Devices

#### 3.1.1. Effect of GeSi Thicknesses on NS Release in Stacked GeSi/Si Samples

**Figure 2:**
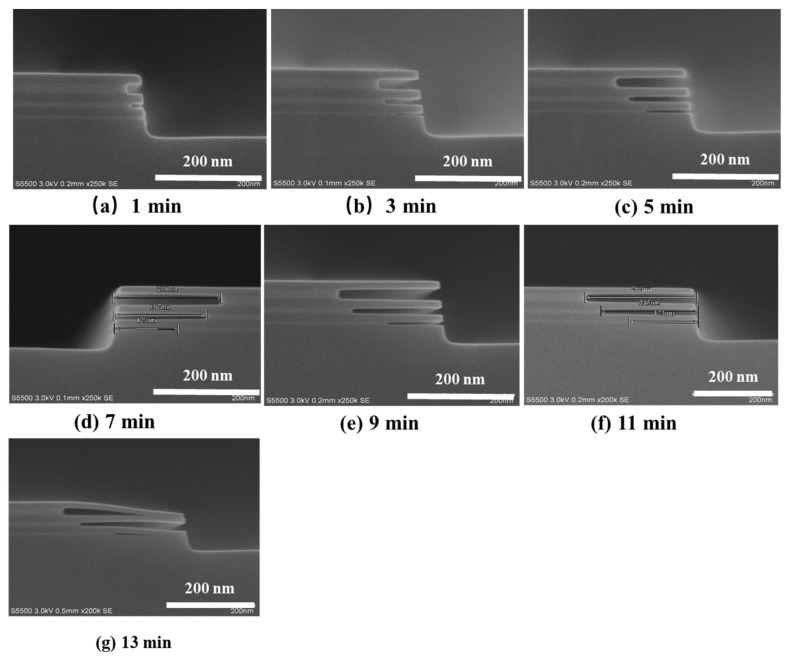
Scanning electron microscopy (SEM) images of GeSi/Si stacks after different etching times: (**a**) 1 min, (**b**) 3 min, (**c**) 5 min, (**d**) 7 min, (**e**) 9 min, (**f**) 11 min, and (**g**) 13 min, respectively.

The thickness of the GeSi layer in GeSi/Si stacks determines the final spacing of stacked GAA Si NW/NS channel MOSFETs, which affects the channel morphology and the filling characteristics of HK/MG stacks [17,18]. In order to study the etching rate of GeSi layers and the etch selectivity of GeSi to Si, GeSi/Si stacks with different thicknesses of the GeSi layer (5 nm, 10 nm, and 20 nm) and 20 nm Si layer as the interlayer between GeSi layers were designed and fabricated. Figure 2 shows SEM images of GeSi/Si stacks samples after different etching times (1 min, 3 min, 5 min, 7 min, 9 min, 11 min, and 13 min) in the mixed corrosion solution (the solution storage for 24 h). As can be seen from the images, a high selective ratio of etching GeSi (5 nm, 10 nm, and 20 nm GeSi in thickness) layer to Si layer is achieved, and the thicker the GeSi is, the faster the etching rate is.

**Figure 3:**
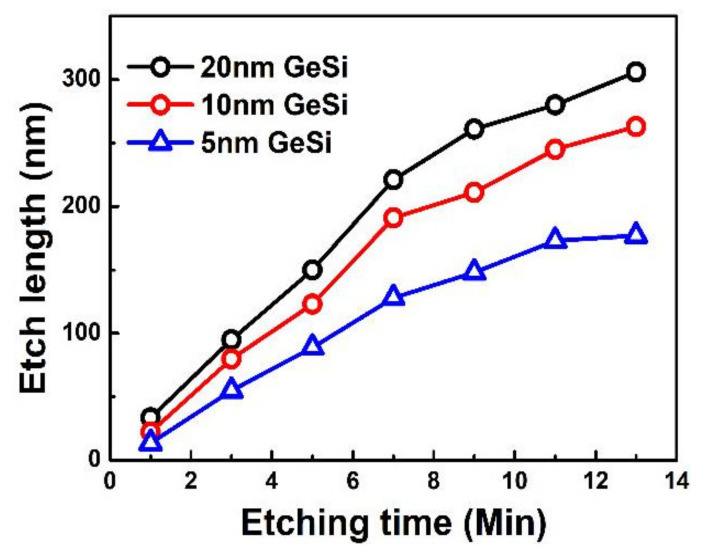
Etch lengths of GeSi layer as a function of etching times for GeSi/Si samples with different GeSi thicknesses.

Figure 3 shows the horizontal etching lengths of 5 nm, 10 nm, and 20 nm GeSi layers as a function of etching time. The etching rate of three-layer GeSi layer becomes slower with the increase of corrosion time after 7 min, and the etching rate of thinner GeSi layer (5 nm) is much easier to achieve a saturate etching rate. Furthermore, the etching rate is much lower for thinner GeSi layer compared with that of thicker GeSi layer. If the etching length of GeSi layer is too long, the Si NSs are easy to “stick” together (see Figure 2g). The thinner GeSi layer may have suffered from a larger force of surface tension of liquid, so it is more likely to be an adhesion effect [10].

#### 3.1.2. Effect of Thermal Anneal on NS Release in Stacked GeSi/Si Samples

**Figure 4:**
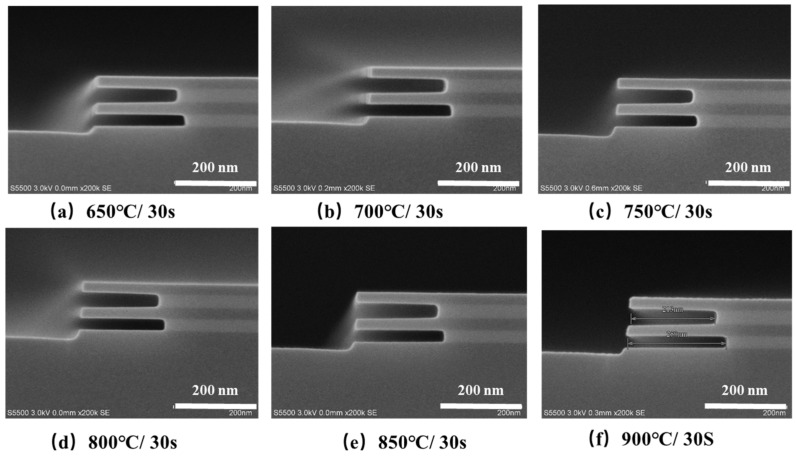
SEM images of the etched stacked GeSi/Si samples after rapid thermal annealing (RTA) at different temperatures: (**a**) 650 °C/30 s, (**b**) 700 °C/30 s, (**c**) 750 °C/30 s, (**d**) 800 °C/30 s, (**e**) 850 °C/30 s, and (**f**) 900 °C/30 s.

During the fabrication of stacked GAA Si NW/NS MOSFETs, a multi-step high temperature annealing process is needed, such as shallow trench isolation (STI) annealing and source drain (SD) activation. The high temperature processes would result in a fast atom diffusion in multi-layer GeSi/Si stacks, and the abrupt interfaces among the multi-layer GeSi/Si stacks layers would be destroyed, which would affect the structure, morphology, and quality of the formed Si NS channels [19,20,21]. In order to study the influence of annealing temperature on the GeSi selective etch, the samples with GeSi/Si stack arrays were annealed at 650 °C, 700 °C, 750 °C, 800 °C, 850 °C, and 900 °C for 30 s, respectively, in N_2_ atmosphere using rapid thermal annealing (RTA) equipment. Then, all the annealed samples were put into the wet-etching solution (the solution hold on 24 h) for the same 8 min. Figure 4 shows the SEM images of the GeSi/Si samples with different annealing conditions after 8 min wet-etch. As can be seen from the images, all samples achieved high selective etching ratio of GeSi to Si below 900 °C, but the etching lengths of the GeSi layers were different.

**Figure 5:**
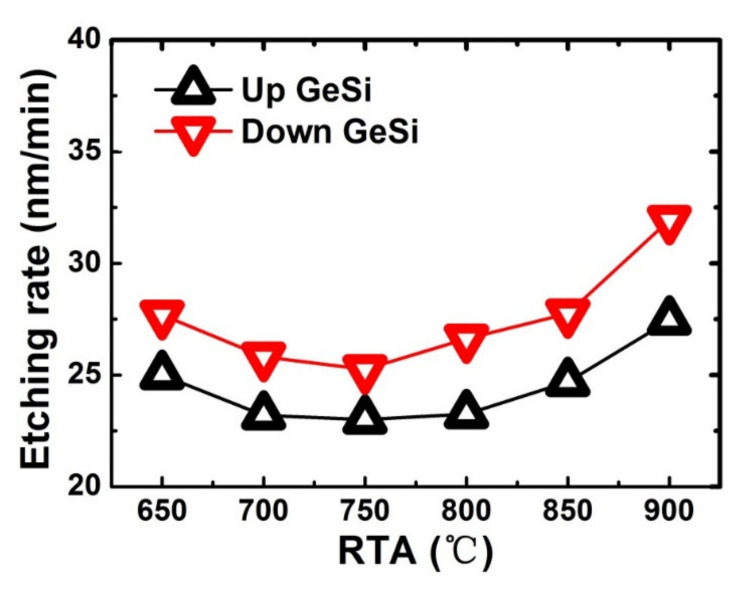
The etching rates of the top and bottom GeSi layers as a function of different RTA temperatures.

Figure 5 shows the corresponding etching rates of the top and bottom GeSi layers. The etching rates are achieved by calculated ratio of the measured etching lengths to etching times. As can be seen from the images, the etching rate of the samples first decreases and then increases with the increase of RTA temperatures, exhibiting a “U” shape curve. The etching rate of GeSi layer is the slowest when the samples were annealing at 750 °C compared with that of other samples at higher or lower annealing temperatures. As the epitaxial growth temperature of GeSi/Si stacks is about 725 °C, annealing at higher temperature or lower temperature processes may result in partial stress release of the GeSi/Si stacks, which may produce defects at the GeSi/Si interface and increase the etching rate of GeSi layer. The remaining Si layer thickness becomes thinner and the etched finger-shaped Si sheets exhibit little “warpage” at 900 °C/30 s, which may be caused by the inter-diffusion at GeSi/Si interface and the stress release of the GeSi/Si stacks, as is shown in Figure 5f.

### 3.2. Device Fabrication and Structure Characterization

#### 3.2.1. Process Monitoring of the Stacked Si NS Devices at Different Fabrication Stages

**Figure 6:**
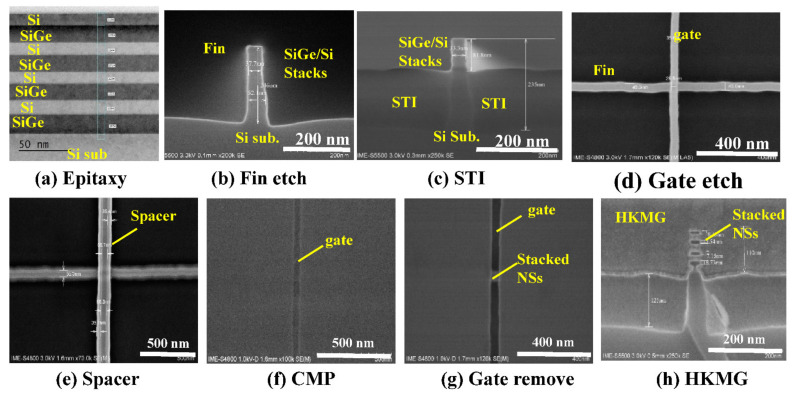
SEM and transmission electron microscopy (TEM) images of stacked GAA NS devices at different fabrication stages: (**a**) epitaxy of multi-layer GeSi/Si stacks, (**b**) fin etch by reactive ion etching (RIE) process, (**c**) STI formation, (**d**) dummy gate formation, (**e**) spacer formation, (**f**) poly gate open by CMP, (**g**) dummy gate removal, and (**h**) Si NS release and HK/MG fill.

Figure 6 shows the SEM and TEM images of the stacked Si NS devices during different fabrication steps. Figure 6a shows the TEM image of multi-layer GeSi/Si stacks with good uniformity, and the thicknesses of GeSi and Si layers are 12 nm and 10 nm, respectively. Very sharp fin with high aspect ratio is achieved by advanced dry etch process (see Figure 6b). 

Figure 6c shows the cross-sectional SEM image of the device after shallow trench isolation (STI) formation. As can be seen from the images, the STI is well controlled with very flat surfaces and the multilayer GeSi/Si stacks on the top of the fin are revealed. Figure 6d shows the top view SEM image of devices after gate formation, which is ~30 nm in width. Conformal spacers are observed at two sides of dummy gate, which are used as self-aligned mask for SD doping (see Figure 6e). A top view of stacked GAA Si NS devices after dummy gate CMP is shown in Figure 6f. The surface of ILD0 layer is relatively smooth, and a ~30 nm poly gate is revealed. Figure 6g shows a top view of the 30-nm-*L*_g_ device after dummy gate removal and Si NSs release. The α-Si dummy gate was entirely removed, and the stacked Si NS channels appear in the open gate trench. Figure 6h shows the cross-sectional SEM image of the stacked GAA Si NS channels after deposition of multilayer ALD HK/MG stacks. As can been seen from the image, very uniform 4-layer stacked NS channels are formed and well surrounded by HK/MG stacks.

#### 3.2.2. Structure Characterization of the Stacked GAA Si NS Devices

**Figure 7:**
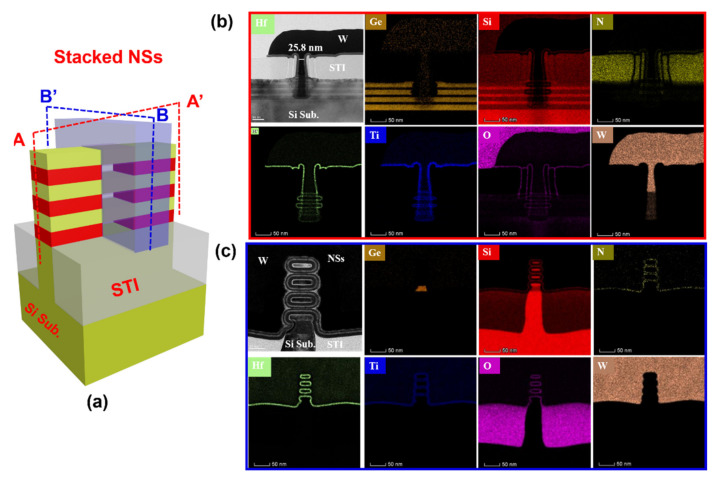
(**a**) A schematic of the stacked GAA Si NSs device, and (**b**,**c**) show cross-sectional TEM images along the direction of AA’ (cut across the fin top) and BB’ (cut across the channel direction), and the corresponding energy-dispersive X-ray spectroscopy (EDX) maps for Ge, Si, N, Hf, Ti, O and W distribution, respectively.

Figure 7a shows a schematic of the stacked GAA Si NSs device, and Figure 7b,c are cross-sectional TEM images along different directions (AA’ cut across the fin top and BB’ cut across the channel direction) and corresponding EDX maps for O, N, Si, Hf, Ti, and W distribution, respectively. As can be seen from Figure 7b, the physical *L*_g_ is 25.8 nm and the stacked GAA Si NSs device is well fabricated, because the SD fin, spacer, and gate trench are well protected with conformal ILD0 material, allowing the Si NS channels and conformal HK/MG GAA structure to be preserved after the final process steps. As can be seen from Figure 7c, there are four uniform stacked Si NS channels formed and thickness of the NSs is about 6 nm, implying the well-controlled Si NS release and fabrication processes [22,23,24]. The Si NSs channel were surrounded by the conformal ALD multilayer HKMG stacks to form GAA structure, which could provide a good gate control ability to the ultrathin Si NS channels. However, there is residual of the bottom GeSi layer for stacked GAA Si NS channel, which deteriorates the gate control ability and electrical characteristics of the devices [25].

#### 3.2.3. Si NS Channels Release Control with Different Etching Processes

**Figure 8:**
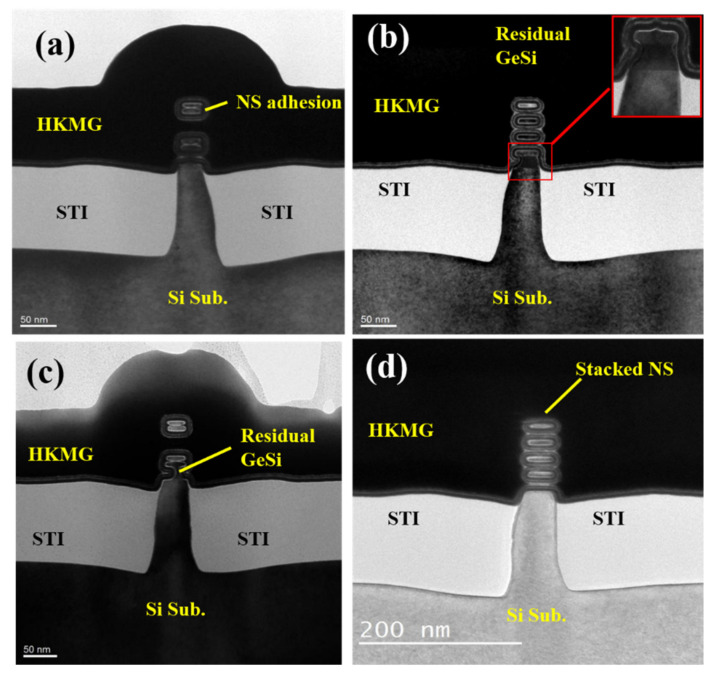
Cross-sectional TEM images of stacked GAA Si NS channel with (**a**) NS channels sticking to each other, (**b**) the residual of the bottom GeSi, (**c**) NSs channels sticking to each other and a thin residual of the bottom GeSi, and (**d**) complete NS release.

In the actual fabrication of the stacked GAA Si NS devices, there is the complex integration approach, variation of process fluctuation, and Si NS channels releasing process control, resulting in the non-ideal stacked GAA Si NS channels [26,27]. In the NSs release processes of stacked Si NS channels, the NS channels are easy to stick to each other, especially in the case of wet etching approach with a large interfacial tension, as shown in Figure 8a. As can be seen from the image, the upper two NSs are connected together, and the bottom two NSs are also connected together, which affects the filling of multi-layer HK/MG stacks and the gate control characteristics of the devices. Figure 8b shows the cross-sectional TEM images of stacked GAA Si NS channels with the residual of the bottom GeSi, which may be caused by a higher height of the STI preventing the bottom GeSi from etching. The non-ideal structure with the residual of the bottom GeSi introduces the parasitic channels at the bottom of the channel and deteriorates the gate control ability of the devices [27]. There is also the phenomenon of NS channels sticking to each other and to the residual of the bottom GeSi in the NSs channels, which greatly degrades the performance of the devices, as shown in Figure 8c. Figure 8d shows the cross-sectional TEM images of stacked GAA Si NS channels with the complete NS release by controlling the Si NS release process. As can be seen from image, the device’s structure without the residual bottom GeSi could form the ideal GAA Si NSs structure with conformal ALD HK/MG stacks.

### 3.3. Influence of GP Doping on Performance of Devices

#### 3.3.1. Experimental Results of GP Doping on Performance of Devices

**Figure 9:**
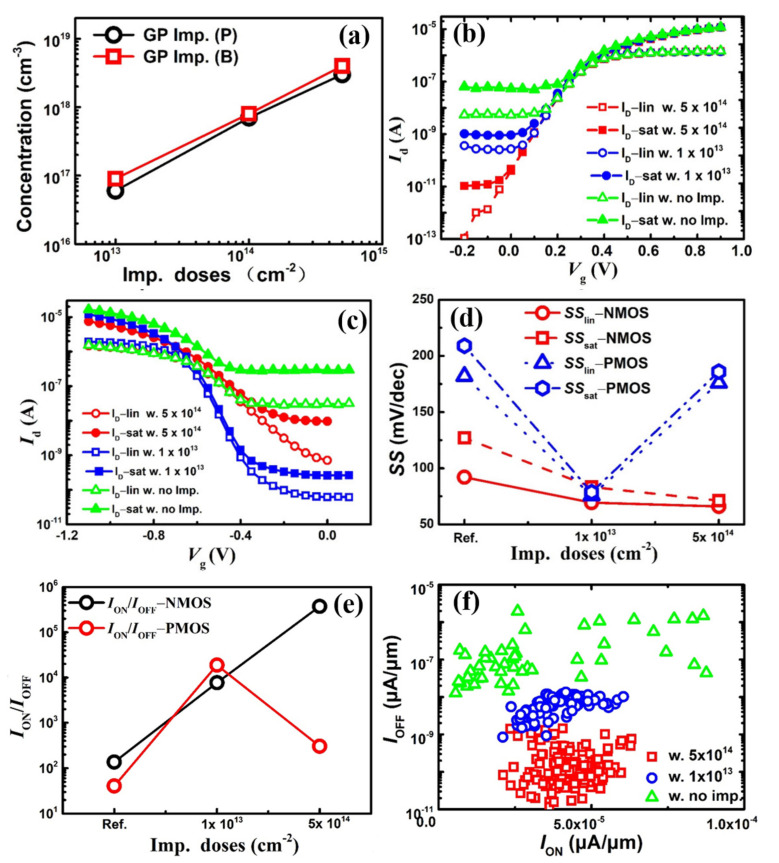
(**a**) Concentration of dopants as a function of doping doses of implant doping doses, (**b**,**c**) experimental *I*_D_-*V*_G_ of *n-*/*p-*type stacked GAA Si NS devices, (**d**,**e**) *I*_ON_/*I*_OFF_ ratios, *SS*_lin_ and *SS*_sat_ extracted with different GP doping conditions, and (**f**) *I*_ON_-*I*_OFF_ mapping of *n-*type stacked GAA Si NSs devices.

In this part, an effective approach of the GP doping was selected to suppress parasitic sub-fin channels of the 4-layer stacked GAA Si NSs devices based on FinFET fabrication flow. The simulated doping concentrations as a function of the P and B doping doses (1 × 10^13^ cm^−2^, 1 × 10^14^ cm^−2^, and 5 × 10^14^ cm^−2^) are shown in Figure 9a. There is approximately a linear relationship between doping concentrations and doses, and the P (B) doping concentrations are approximately 6 × 10^16^ (9 × 10^16^) cm^−3^, 7 × 10^17^ (8 × 10^17^) cm^−3^, and 3 × 10^18^ (4 × 10^18^) cm^−3^, respectively. Figure 9b,c show the typical experimental transfer curves of *n-/p*-type stacked GAA Si NSs devices with no implantation, 1 × 10^13^ cm^−2^ and 5 × 10^14^ cm^−2^, respectively. As can be seen from the images, the drain currents are almost the same at on-state, but there is a great influence on the leakages at off-state both for *n*-type and *p*-type stacked GAA Si NSs devices, respectively. The leakages are reduced by approximately two orders of magnitude for *n*-type GAA Si NSs device by using a GP doping P dose of 1 × 10^13^ cm^−2^. However, there are approximately over three orders of magnitude for *p*-type with a GP doping B dose of 1 × 10^13^ cm^−2^. There are approximately another two orders of magnitude reduction in leakages for *n*-type with a GP doping dose of 5 × 10^14^ cm^−2^ P compared to those with 1 × 10^13^ cm^−2^ (see Figure 9c). However, the leakage increases approximately two orders of magnitude for *p*-type with a GP doping dose of 5 × 10^14^ cm^−2^ B compared to those with 1 × 10^13^ cm^−2^ (see Figure 9b). The values of *I*_ON_/*I*_OFF_ ratios of *SS*s are extracted from the transfer curves with different GP doping conditions, as is shown in Figure 9d,e, respectively. The *I*_ON_/*I*_OFF_ ratios of *n*-type devices increase with the increase of GP doping doses. However, the *I*_ON_/*I*_OFF_ ratios of *p*-type devices exhibit first increases and then decreases with the increase of GP doping doses. Meanwhile, the values of *SS*_lin_ and *SS*_sat_ decrease with the increase of GP doping doses for *n*-type devices, but the *p*-type devices also exhibit a trend of faster decreases and then increases with the increase of GP concentrations (see Figure 9e). The results show that the *n*-type and *p*-type stacked GAA Si NSs devices need different GP doping conditions, and the optimization of parasitic channel for *p*-type devices is more difficult and complex than that of *n*-type devices. The *I*_ON_-*I*_OFF_ mapping of *n*-type stacked GAA Si NS devices with different GP doping conditions is shown in Figure 9f. The current density of *I*_ON_ and *I*_OFF_ is normalized according to the width of the Si nanosheets. As can been seen from the images, the off-current of the device is reduced by four orders of magnitude compared with that of without GP doping.

#### 3.3.2. Simulation Results of GP Doping on Performance of *p*-Type Devices

**Figure 10:**
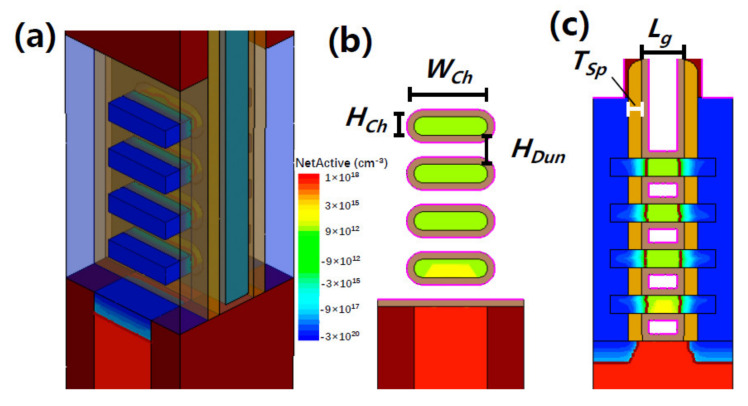
(**a**) Simulated GAA Si NS device’s structure and cross-sectional images (**b**) perpendicular to and (**c**) along the channel direction.

In order to further study the influence of GP doping on the suppression of parasitic channels and seek out the optimal conditions for *p*-type stacked GAA Si NSs devices application, 3D TCAD simulations on the stacked GAA Si NSs devices, including process and device simulations, were carried out using Sentaurus TCAD tools. Figure 10 shows the schematic of device structure adopted in this paper similar to the real structure of the fabricated devices, and the stacked GAA Si NS device comprises 4 stacked NSs channels (see Figure 10b,c). Table 1 summarizes the parameters used in the simulation, and ultra-scaled 2 nm node device with 12-nm-*L*_g_ and 4-layer stacked 6-nm-thick Si NSs channels were adopted according to the International Roadmap for Devices and Systems (IRDS) [28]. Hurkx Band-to-Band model as well as Fermi-Dirac statistics, Shockley-Read-Hall, and Anger recombination models were implemented in the device simulation [29].

**Figure 11:**
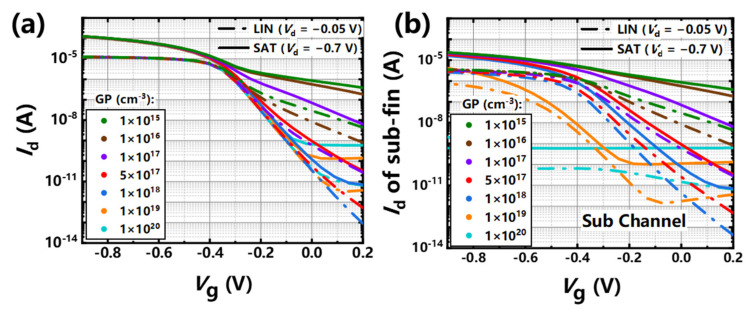
*I*_d_-*V*_g_ curves of 12-nm-*L*g, (**a**) NS-FETs and (**b**) sub-channel with different GP doping concentrations.

The transfer curves of 12-nm-*L*_g_ *p*-type stacked GAA Si NSs devices with different GP concentrations are shown in Figure 11a. 

As can be seen from the image, there are large leakages at a GP doping concentration of 3 × 10^18^ cm^−3^ and the leakages get smaller with the increase of GP doping concentrations. However, the leakage becomes more serious and exhibits obvious gate-induced drain leakage (GIDL) effect when the GP concentration reach 1 × 10^19^ cm^−3^. In order to study and analyze the detailed reasons of the phenomenon, the corresponding changes of *I*_d_-*V*_g_ curves of the parasitic sub-fin (by excluding the current of the NS channels) under different GP concentrations are shown in Figure 11b. The leakage current of parasitic sub-fin channel at the bottom shows two trends with the increase of doping concentrations: (1) fast decrease of the off-state current; and (2) shift of the *V*_th_ to the negative direction. The leakage of the sub-fin at off-state has no obvious change with the decrease of *V*_g_ with the GP doping dose of 1 × 10^15^ cm^−2^, which is caused by a large punch through current from sub-fin and deteriorates the device’s performance. With the increase of GP doping concentrations, the leakage current of the sub-fin at the off-state decreases exponentially and the values of *V*_t_ shift become a little larger, which is more helpful to reduce the off-state leakage of the whole device. There is a large *V*_t_ shift (~200 mV) of the *I*_d_-*V*_g_ curves of the sub-fin at GP doping concentration of 1 × 10^19^ cm^−3^ compared with that of with a GP doping concentration of 1 × 10^18^ cm^−3^. However, the off-state leakage of the sub-fin gets larger and the curves exhibit gate induced barrier lowering (GIDL), which may be caused by a larger overlap for *p*-type devices. The leakage current of the sub-fin remains stable and does not change with the increase of *V*_g_s with a GP doping concentration of 1 × 10^20^ cm^−3^, which demonstrates another side-effect of band-to-band tunneling (BTBT) current occurring in degenerate semiconductor *p*-*n* junction at very high GP doping concentrations.

**Figure 12:**
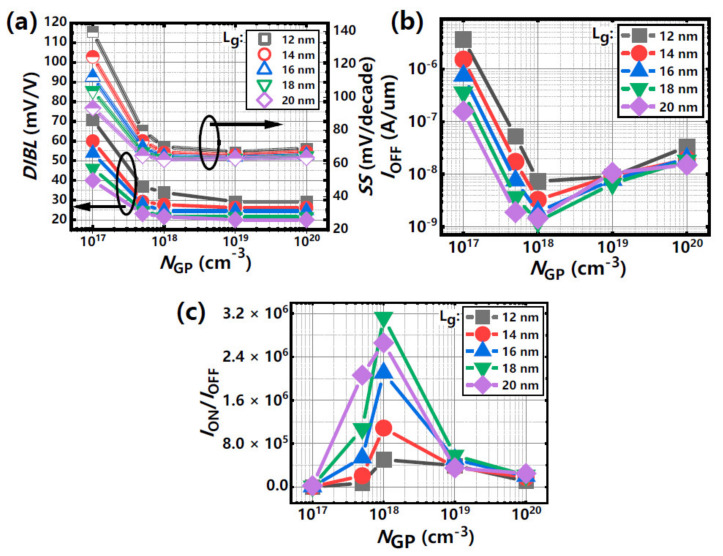
(**a**) *DIBL* and *SS*_sat_, (**b**) *I*_OFF_ and (**c**) *I*_ON_/*I*_OFF_ ratio extracted from devices with different *L*_g_s and GP doping concentration.

In order to further investigate the influence of sub-fin channels with different GP doping concentrations on the performance of stacked GAA Si NSs devices with different *L*_g_s, some key electrical parameters of the devices (e.g., *DIBL*s, *SS*_sat_s, *I*_OFF_, and *I*_ON_/*I*_OFF_ ratios) are extracted and shown in Figure 12.

The current density of the *I*_OFF_ is normalized according to the width of the stacked Si nanosheets. The values of *DIBL*s and *SS*_sat_s as a function of different GP doping concentrations with different *L*_g_s for the stacked GAA Si NS device are shown in Figure 12a. The values of *DIBL*s and *SS*_sat_s have a fast initial decrease with the increase of GP doping concentrations and almost remain stable when GP doping concentrations are over 5 × 10^17^ cm^−3^. Meanwhile, the values of *DIBL*s and *SS*_sat_s increase with the decrease of *L*_g_s and the deteriorated *DIBL*s and *SS*_sat_s could also be improved even when the *L*_g_s is scaled to 12 nm for stacked GAA Si NS devices. In addition, the minimum *I*_OFF_s and the largest *I*_ON_/*I*_OFF_ ratios are achieved at GP doping concentrations of 1 × 10^18^ cm^−3^ for different *L*_g_s and these parameters worsen because of the induced tunneling current for the degenerated semiconductor effect (Figure 12b,c). Furthermore, the electrical conductance in bottom NS channel is degraded for high-concentration-dopant out-diffusion phenomena in this technology. Therefore, the GP doping doses of approximately 1 × 10^14^ cm^−2^ is a better condition for reducing sub threshold leakage and improving *p*-type device performance.

Table 2 shows a system parameters comparison of some relevant reported results in recent years and our fabricated stacked GAA Si NS devices. For the first time, 4-layer stacked GAA Si NS channels with 6 nm in thickness and 30 nm in width were firstly fabricated on bulk substrate and achieved good morphology of *n*-/*p*-type GAA Si NS devices. Furthermore, a larger *I*_ON_/*I*_OFF_ ratio and smaller values of *SS*s and *DIBL*s for 25.8 nm-*L*_g_ devices is achieved for the fabricated stacked GAA Si NS devices by the optimization of suppression of parasitic channels and device’s structure. The results indicated that the stacked GAA Si NS devices fabricated have much better comprehensive characteristics compared with those of the compared devices reported [10,11,12,13,30].

## 4. Conclusions

In summary, the optimization of NS release process of stacked GAA Si NS devices was carried out and achieved optimal GAA Si NS channels. An over 100:1 selective wet-etch ratio of GeSi to Si layer is achieved for GeSi/Si stack samples with different GeSi thickness and annealing temperatures (≤900 °C). Furthermore, the influence of the GP doping in Si sub-fin region to improve electrical characteristics of the devices was extensively investigated by experiments and simulations. The subthreshold characteristics of *n*-type devices are significantly improved with the increase of GP doping doses. However, the *p*-type devices were first improved and then deteriorated with the increase of GP doping doses which demonstrates better electrical characteristics with the GP doping concentrations of 1 × 10^18^ cm^−3^. Furthermore, 4-layer stacked GAA Si NS channels with 6-nm-thick and 30-nm-width were firstly fabricated on bulk substrate, and the performance of the stacked GAA Si NS devices was greatly improved by the optimization of suppression of parasitic channels and device’s structure, which achieved a larger *I*_ON_/*I*_OFF_ ratio and a smaller values of *SS*s and *DIBL*s. Therefore, the optimization of the fabricated stacked GAA Si NS devices approaches and simulated results provide a good guide for its potential mass application in future.

## Figures and Tables

**Table 1:** Parameters defined in stacked GAA Si NS device.

Parameters of GAA Si NS Devices	Values
Type	*p*-type
Stacks of NS channels	4
*L*_g_ (nm)	12
NS thickness (nm)	5
Wideness of NSs (nm)	20
EOT (nm)	1
Spacing between NSs (nm)	8 nm
N_ch_ (cm^−3^)	1 × 10^15^
N_SD_ (cm^−3^)	3 × 10^20^
Thickness of spacers (nm)	4 nm

**Table 2:** A comparison of GAA Si NS devices in recent years.

Ref.	(30 IBM) 2017 VLSI	(11 Samsung) 2018 IEDM	(12 NNDL) 2018 EDL	(13 IBM) 2019 IEDM	(14 CEA Leti) 2020 VLSI	(IMECAS) This Work
Channel	Stacked Si NS	Stacked Si NS	Stacked Ge NS	Stacked Si NS	Stacked Si NS	Stacked Si NS
N/P type	N/P	N/P	N/P	N/P	N	N/P
Fin/Sub.	GeSi/Si stack on Si	-	Ge/Si stack on SOI	SiGe/Si stack on Si	SiGe/Si stack on SOI	SiGe/Si stack on Si
Num. of Stacked NSs	3	-	3	3	7	4
STI Annael	≤900 °C	-	-	≤900 °C	-	≤850 °C
Release	In RMG	In RMG	In RMG	In RMG	In RMG	In RMG
NS forming	SiGe etch	-	Si etch	SiGe etch	SiGe etch	SiGe etch
NS Width/Thickness	~25/6 nm	-	~90/40 nm	-	~30/10 nm	30/6 nm
*L*_g_	12 nm	-	90 nm	12 nm	45 nm	25.8 nm
*SS* (mV/dec)	75(N)/85 (P)	65(N)/67 (P)	140(N)/130(P)	73(N)/74 (P)	64(N)	71.2(N)/78.7 (P)
*DIBL* (mV/V)	32(N)/24 (P)	20(N)/24 (P)	-	32(N)/35 (P)	10(N)	9 (N)/22 (P)
*I*_ON_/*I*_OFF_	~10^4^	~10^5^	~10^4^	~10^6^	~10^5^	3.15 × 10^5^

## Data Availability

The data presented in this study are available on request from the corresponding authors.
